# Aortic Origin Coronary Artery Anomalies: A Case Series

**DOI:** 10.7759/cureus.57755

**Published:** 2024-04-07

**Authors:** Akin Torun, Sahhan Kilic, Mehmet Seker, Volkan Camkiran

**Affiliations:** 1 Department of Cardiology, Sultan II. Abdulhamid Han Training and Research Hospital, Istanbul, TUR; 2 Department of Cardiology, Bahcesehir University Medical Park Goztepe Hospital, Istanbul, TUR

**Keywords:** congenital heart disease, anomalous aortic origin, coronary intervention, coronary artery disease, coronary anomalies

## Abstract

Coronary artery anomalies (CAAs) are rare, but they can cause serious consequences, complicate the diagnosis of coronary artery disease (CAD), and hamper the ability of the physician to perform the correct intervention for patients with CAD. The widespread use of coronary computed tomography and angiography has shown that the incidence is higher than previously thought. CAAs can occur with various anatomical features. We present three rare examples. The first example involves a circumflex artery (CX) that originates from a different ostium on the right side, despite the presence of left arteries in normal anatomical structures. The second case involves an accessory CX originating from the right coronary artery (RCA) ostium, despite the CX origin being in the left cusp. Finally, the third case involves an accessory left anterior descending artery (LAD) originating from the RCA ostium, despite the LAD origin being in the left cusp. There were no high-risk features in all three cases, and no symptoms were observed during follow-up with the patients. The occurrence of these cases is exceptional and may be overlooked; hence, their identification has significance.

## Introduction

Coronary artery anomalies (CAAs) may have a genetic basis, and retrospective studies of CAAs have shown the incidence to be between 0.4% and 1.6% [1]. About 26% of coronary anomalies involve some kind of aortic root abnormality (such as bicuspid aortic valve), at least asymmetry of the aortic sinuses. However, the incidence of CAAs varies widely in the literature. This is likely a reflection of referral bias and the variability in definitions of “anomalous” and “normal variant.” According to the literature, CAAs affect around 1% of the general population, ranging from 0.3% to 5.6% in studies on patients undergoing coronary angiography, and in approximately 1% of routine autopsy [2]. Neither of these figures may be truly representative, the incidence of coronary anomalies in necropsy patients may be biased by cause of death, and angiography is usually performed because of the suspicion of ischemia. CAAs might be asymptomatic but can cause symptoms such as syncope, chest pain during physical activity, arrhythmia, and even serious consequences such as sudden cardiac death (SCD). CAAs can hamper the diagnosis of coronary artery disease (CAD) and the effective and quick performance of the intervention, especially in cases of acute coronary syndrome [3]. Some anatomical features are risky; CAA with acutely angled take-off from the aorta resulting in a slit-like orifice with the reduced lumen and anomalous coursing between the aorta and the pulmonary artery is associated with the greatest risk for SCD. It should also be noted that CAA may accompany other congenital cardiac anomalies. Detecting myocardial ischemia in patients with CAA is the most critical step. Identifying potential rare cases holds crucial significance. Therefore, we present uncommon CAA cases in our article.

## Case presentation

Case 1

**Figure 1:**
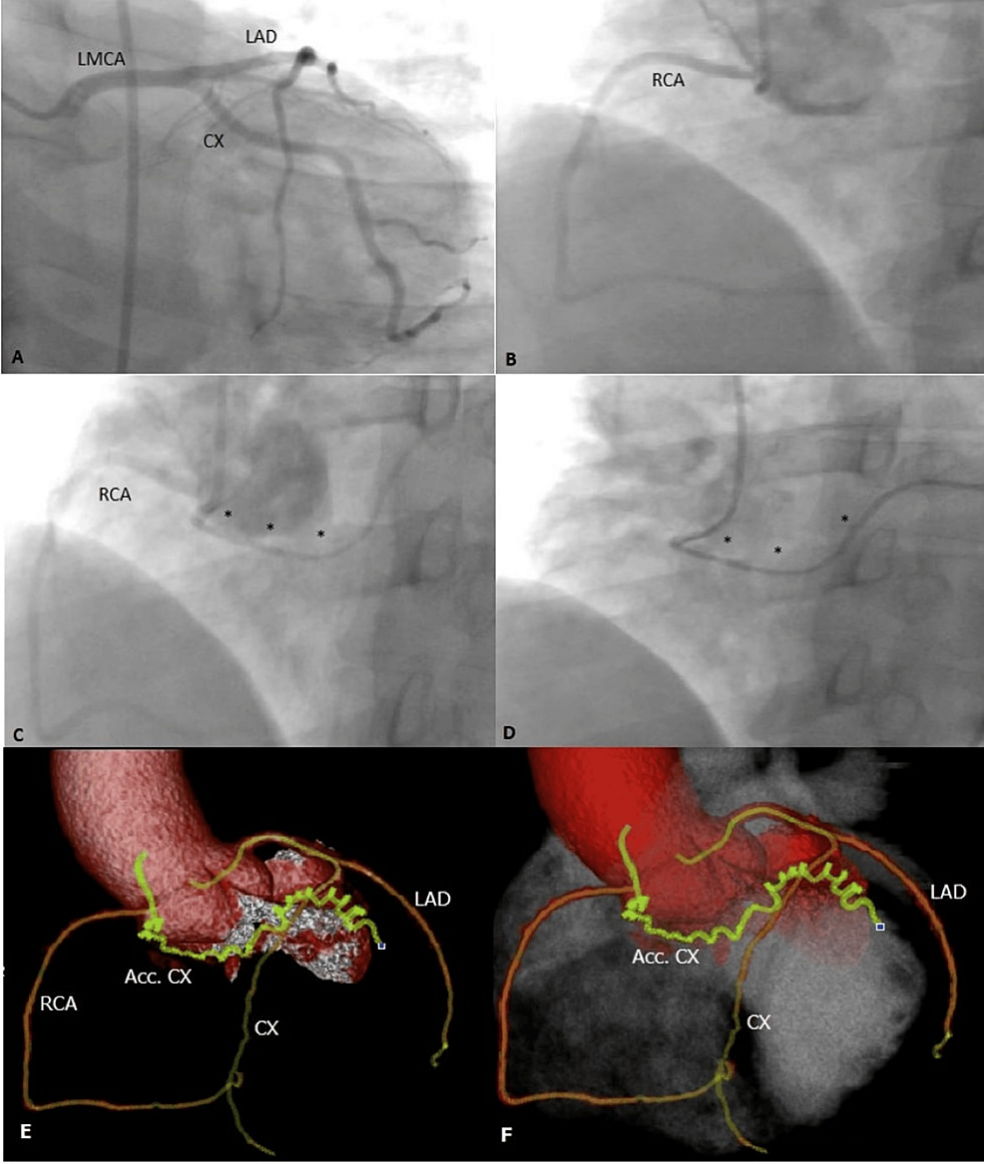
Case 1 A) Normal course of LMCA, LAD, and CX, AP caudal view. B) Normal course of RCA, RAO view. C) RCA and left-course accessory coronary artery shown by cusp injection, modified RAO view. D) Accessory artery originates from the right and course to the left, modified RAO view. E/F) Accessory coronary artery originating from the right and normal coronary arterial anatomy. LMCA, left main coronary artery; LAD, left anterior descending artery; CX, circumflex artery; AP, anteroposterior; RCA, right coronary artery; RAO, right anterior oblique.

A 59-year-old male who had a history of hypertension and hyperlipidemia presented to the hospital with a gradual onset of shortness of breath. Angiography was planned due to the complaint of chest pain during the treadmill exercise test. The left main coronary ostia was cannulated with a 5 F Judkins left 4 diagnostic catheter with no anatomical anomaly or atherosclerotic vascular disease. We clearly observed the course of the left anterior descending (LAD) and circumflex (CX). Then we tried to cannulate the right coronary artery (RCA) with a 5 F Judkins right 4 diagnostic catheter, but this engaged another coronary artery without RCA. This accessory coronary artery was arising from the right coronary sinus and coursing to the distal CX area. Finally, we cannulated the RCA with the same catheter in the normal origin of RCA. RCA was not dominant, and we observed no atherosclerotic lesion. The accessory artery ostium was next to the RCA ostium (Figure 1).

After the invasive coronary angiography (ICA), we decided to attempt coronary computed tomography angiography (CCTA) for the detection of possible abnormalities and accessory arteries. CCTA showed a result consistent with ICA. There was no other anomaly or relationship with the surrounding tissues. The patient did not have any additional symptoms in the follow-up.

Case 2

**Figure 2:**
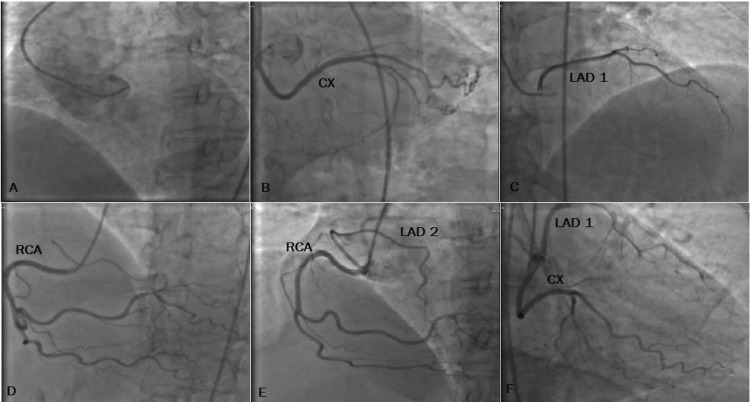
Case 2 A) No coronary ostium was observed during left cusp injection, left caudal view. B) CX originating from the right cusp, AP caudal view, AP caudal. C) LAD originating from the right cusp, modified AP cranial. D) Normal course of RCA, RAO. E/F) Right cusp origin RCA and accessory LAD, RAO and right caudal view. CX, circumflex artery; AP, anteroposterior; LAD, left anterior descending artery; RCA, right coronary artery; RAO, right anterior oblique.

A 54-year-old female with no significant past medical history presented to the emergency department complaining of substernal chest pain for 10 days. Coronary angiography was performed on the patient, who complained of chest pain in the treadmill stress test. No coronary artery originating from the left coronary sinus was observed in the patient during the procedure. Two arteries compatible with the LAD course were observed in the patient. The accessory branch compatible with LAD anatomy originated from a different ostium close to the RCA ostium. Additionally, LAD and CX originated from different ostia (Figure 2). There was no other anomaly. The patient is being followed up with medical treatment and has no complaints at the six-month follow-up.

Case 3

**Figure 3:**
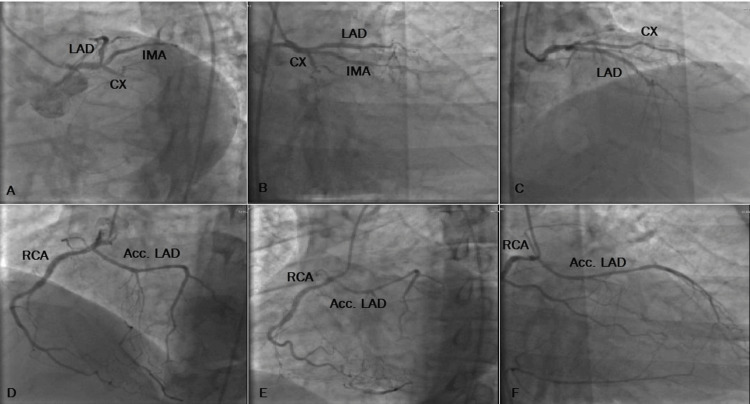
Case 3 A/B/C) LAD is rudimentary after mid region and CX occluded from the proximal segment (left caudal, right caudal, and AP cranial views, respectively). D/E/F) Accessory LAD originates from the proximal segment of the RCA (modified RAO poses and RAO pose, respectively). LAD, left anterior descending artery; CX, circumflex artery; AP, anteroposterior; RCA, right coronary artery; RAO, right anterior oblique.

A 71-year-old male patient with a past medical history of diabetes mellitus and hypertension was admitted to the emergency service with palpitation and epigastric pain. Ventricular tachycardia was detected on his monitor, and cardioversion was performed. Ejection fraction (EF) was 35% on echocardiography, and hypokinetic segments were seen in the inferoapical and lateral segments. LAD was observed as rudimentary after mid-region, and CX was occluded from the proximal segment in left system imaging. RCA was seen with a natural course on the right system imaging, and an accessory LAD was observed with a course to the LAD mid and distal segment (Figure 3). CX was revascularized. In the pre-discharge control echo, EF was 55%, there was a mild hypokinetic segment on the lateral wall, and mild mitral insufficiency was observed. There were no other cardiac anomalies. The patient was discharged with medical treatment. He had no additional complaints during the follow-up.

## Discussion

The widespread use of CCTA has yielded further insight into the epidemiological boundaries of CAAs, the prevalence of which appears to be even higher [4]. CAA has an incidence rate of 2.6-7.9% in an unselected patient population upon CCTA [4,5]. This suggests that the true prevalence of coronary anomalies in the general population may have been underestimated based on ICA. Therefore, case examples that will contribute to the scientific literature on CAA will be valuable.

Yesilyurt et al. showed that the high take-off RCA and LAD, the RCA arising from the left sinus, the LAD arising from the right sinus (RS), and CX arising from the RS are the most commonly seen anomalies and may be considered when CAA is suspected [5]. More rarely, a single ostium from the right or left coronary sinus and an additional accessory artery may be present. However, these anomalies can vary, and it is difficult to selectively engage the artery; in these cases, aortography, CCTA, or magnetic resonance imaging (MRI) can be performed [6]. Additional coronary anomalies should be considered in patients with abnormal aortic origin. Anomalous origin from the pulmonary artery, coronary artery fistula, intramyocardial courses, and possible septal defects should be ruled out first [7]. In one of our cases, we observed LAD, CX, and RCA. The presence of a fourth artery can easily be overlooked in such a situation. It is highly difficult for the cardiologist to consider the existence of the fourth coronary artery unless there is a gap in the myocardial blush. CCTA and MRI may be more effective in such cases.

Congenital CAAs are not common anomalies, but they are nevertheless the second most common cause of SCD among young athletes [8]. Only one-third of patients have symptoms. Chest pain, exertional syncope, or SCD may present as the first signs [9]. When a life-threatening event occurs in patients with CAA, myocardial ischemia should be considered as the primary cause. Therefore, it is important to clarify the suspicion of ischemia and inform the patient.

The prognostic significance of the coronary arteries, particularly the abnormal aortic origin originating from the contralateral sinus, is more remarkable [10]. An interarterial course should be considered to be high risk and is often associated with other anatomical features. However, the subpulmonic, prepulmonic, retroaortic, and retrocardiac course can generally be predicted to not cause SCD [11]. CAA with acute angled take-off from the aorta resulting in a slit-like orifice with reduced lumen and anomalous coursing between the aorta and the pulmonary artery is associated with the greatest risk for SCD. Participation in most competitive sports with a moderate and high cardiovascular demand among individuals with CAA with an acutely angled take-off or an anomalous course between the large vessels is not recommended [12]. CAAs may increase the risk of SCD by causing myocardial ischemia during vigorous exertion, especially in athletes and healthy young people [13]. Moderate and highly intense sports may be considered in individuals who do not have high-risk anatomy and in whom myocardial ischemia has been ruled out.

Suspicion should be the first element in CAA management. The second step should be to detect both CAAs and additional cardiac anomalies with appropriate imaging techniques. The presence of myocardial ischemia is particularly critical to prevent SCD. Asymptomatic young patients without signs of ischemia should be informed. If ischemia is not induced and arrhythmia is not observed in maximal stress tests in asymptomatic patients without anatomical risk, competitive sports may be considered.

## Conclusions

CAA anomalies are not common, but it is crucial to identify them, as in some cases they can cause a severe reduction of blood flow to the myocardium (ischemia) and lead to chest pain, arrhythmias, and SCD, which in themselves can increase the risk of routine procedures. The aim of this article is to provide a relevant and concise overview of CAA and to increase experience with the anomalies through the cases presented.
